# 3,3′-Di-O-methylellagic Acid Isolated from *Euphorbia humifusa* Willd Suppresses Prostate Cancer Cell Viability via Regulating VDAC1 Protein Expression

**DOI:** 10.3390/ph19050652

**Published:** 2026-04-22

**Authors:** Gulijikere Kuerban, Xinling Wang, Chengjing Shen, Mireguli Abulimiti, Jialu Hu, Zilala Yalihong, Aikebaier Maimaiti

**Affiliations:** College of Pharmacy, Xinjiang Medical University, Shang De North Road, Shui Mo Gou District, Urumqi 830017, China; 3257566818@qq.com (G.K.);

**Keywords:** 3,3′-di-O-methylellagic acid, prostate cancer, VDAC1, apoptosis, molecular mechanism

## Abstract

**Background**: Prostate cancer (PCa) is the leading male urinary malignancy globally. Our previous article demonstrated the anti-PCa activity of Euphorbia humifusa Willd water extract (EHW) and some of its compounds via downregulating AR expression, but the anti-PCa active compounds from Euphorbia humifusa Willd (EH) and their mechanisms of action are yet to be clarified. Thus, the current article studied the in vitro anti-PCa effects of 3,3′-di-O-methylellagic acid (3,3′-di-O-Me-EA) derived from EHW and the related mechanism involved. **Methods**: 3,3’-di-O-Me-EA was isolated from EHW applying bioassay-guided fractionation. The spectroscopic methods were used to determining the structure of 3,3′-di-O-Me-EA. The drug-likeness and ADMET properties (absorption, distribution, metabolism, excretion, and toxicity) of 3,3′-di-O-Me-EA were analyzed in silico. Molecular docking and real-time surface plasmon resonance (SPR) analysis were performed to measure the interaction of 3,3′-di-O-Me-EA and VDAC1 protein. The viability and apoptosis of 22RV-1 and DU145 PCa cells were determined using MTT and Annexin V-FITC staining assay, respectively. q-PCR and Western blot experiments were used to analyzing the gene and protein expressions of VDAC1. **Results**: 3,3′-di-O-Me-EA was isolated and purified from EHW with a purity of ≥90.06%, and its structure was identified by HRTOF mass, NMR, and an authentic standard. In silico ADMET analysis indicated its favorable drug-like and pharmacokinetic properties. Molecular docking and SPR results confirmed that 3,3′-di-O-Me-EA could bind with the VDAC1 protein. Moreover, 3,3′-di-O-Me-EA dose- and time-dependently inhibited 22RV-1 and DU145 PCa cell viability, and induced apoptosis in a dose-dependent manner (*p* < 0.05). RT-qPCR and Western blot results showed that 3,3′-di-O-Me-EA dose-dependently up-regulated VDAC1 gene and protein expression levels in 22RV-1 and DU145 cells (*p* < 0.05). Meanwhile, in VDAC1-depleted 22RV-1 and DU145 cells, 3,3′-di-O-Me-EA down-regulated VDAC1 gene and protein expression levels, increased cell viability, and inhibited apoptosis compared to 22RV-1 and DU145 cells (*p* < 0.05). Furthermore, 3,3′-di-O-Me-EA enhanced VDAC1 gene and protein expression levels, inhibited cell viability, and induced apoptosis in VDAC1-overexpressed 22RV-1 and DU145 cells compared with 22RV-1 and DU145 cells (*p* < 0.05). Overall, EH active compound 3,3′-di-O-Me-EA may inhibit viability and induce apoptosis of 22RV-1 and DU145 PCa cells via up-regulating VDAC1 gene and protein expression levels. **Conclusion**: The results indicated that the 22RV1 and DU145 PCa cell viability inhibitory effects of 3,3′-di-O-Me-EA isolated from EH may be mediated by induction of apoptosis through up-regulation of VDAC1 gene and protein expression levels.

## 1. Introduction

PCa is a highly prevalent malignancy in men [1], ranking as the second leading cause of male cancer-related death [2], and the second most common solid organ cancer in the world [3]. The global cancer statistics reported that approximately 1.4 million new cases and 375,000 deaths occurred in 2020 worldwide [4]. In recent years, the incidence of PCa has significantly increased in China, as well as globally, due to factors such as an aging population, changes in lifestyle, diet, hormonal levels, and genetic predisposition [5,6], and it has become a major threat to men’s health [7]. Although PCa is often diagnosed at an early stage, the risk-benefit ratio of treatment remains uncertain. Different treatment modalities, including surgical resection, endocrine therapy [8], external beam radiotherapy [9,10], brachytherapy [11,12], chemotherapy, hormonal therapy [13], and immunotherapy [14] show good efficacy in the early stages of PCa. However, over 30% of patients experience recurrence and metastasis [15], and most treatments are associated with severe side effects. Therefore, it is necessary to discover and develop novel therapeutic agents that are highly effective, inexpensive, and have low side effects.

Folk medicinal herbs provide natural bioactive compounds for the treatment of various cancers [16,17,18]. Many plant extracts offer remarkable health benefits with low side effects and delay drug resistance [19]. EH belongs to the Euphorbia genus of the Euphorbiaceous family [20]. It is widely distributed in all parts of China and is commonly used for the treatment of diarrhea, jaundice, dysentery, enteritis, diabetes, and asthma for a long time [21,22]. Numerous studies have reported that the main active constituents of EH, namely, flavonoids, phenols, alkaloids, triterpenoids, phytosterols, and sesquiterpenoids, exhibit various pharmacological properties [23,24,25], such as anti-inflammatory [21], anti-viral [26], hypoglycemic [27], and anti-tumor activities [28]. Among them, the anti-tumor activity of this plant has drawn the particular interest of some researchers [29]. As demonstrated by Shin SY et al. [30], the EH ethyl acetate fraction significantly inhibits breast cancer MDA-MB-231 cell invasion and migration and effectively reduces lung metastasis of mouse 4T1 breast cancer cells. In addition, it has been reported that EH-quercetin can inhibit the metastasis of the breast cancer MDA-MB-231 cells [31]. Another article also documented that EH-arbutin induces apoptosis in breast adenocarcinoma MCF-7 cells through p53 and Caspase 3 activation [28]. Wang P et al. [32] reported that EH flavanol gavage suppresses cervical cancer tumor growth and activates Caspase-8, Caspase-3, and P16 proteins in vivo. It has been demonstrated that EH and EH-quercetin both inhibit hepatocellular carcinoma H22 cell growth [31,33]. EH gavage as well as EH-kaempferol exert strong anti-tumor effects against colorectal cancer [34,35]. EH-β-sitosterol has been reported to inhibit pancreatic cancer MIA-PaCa-2 and BXPC-3 cell proliferation and migration [36]. EH-GA inhibits T24 human bladder cancer cell growth and induces apoptosis [37]. However, research on its bioactive material basis remains insufficient, especially concerning the isolation of monomeric compounds with anti-PCa activities and their mechanisms of action, which require further exploration [27].

Thus, the current study investigated the in vitro anti-PCa effects of 3,3′-di-O-Me-EA derived from EHW and the related mechanisms involved. We previously demonstrated that EHW contains high amounts of phenolic and flavonoid compounds and significantly inhibits 22RV1 PCa cell viability [38]. Moreover, it was found that EHW up-regulated the VDAC1 protein expression level in both 22RV1 and DU145 cells (Supplementary Figure S1). Based on the above results, we hypothesized that some active compounds from EH may exhibit strong anti-PCa effects via regulating the VDAC1 protein expression. Therefore, we first isolated the 3,3′-di-O-Me-EA from the EHW using bioassay-guided fractionation, and the drug-likeness and toxicity were estimated using in silico ADEMT analysis. Then, the cell viability inhibitory and apoptosis induction effects of 3,3′-di-O-Me-EA were observed in 22RV1 and DU145 cells compared with the VDAC1 inducer Somatostatin (SS). Moreover, the interaction of 3,3′-di-O-Me-EA and the VDAC1 protein was evaluated using molecular docking and SPR analysis. Furthermore, the regulatory effects of 3,3′-di-O-Me-EA on VDAC1 protein expression levels were studied using 22RV1 and DU145 cells, VDAC1-depleted 22RV1 and DU145 cells, and VDAC1 overexpressed 22RV1 and DU145 cells to evaluate VDAC1-mediated cell viability inhibitory and apoptosis induction effects of 3,3′-di-O-Me-EA in PCa cells in vitro.

## 2. Results

### 2.1. Isolation of 3,3′-Di-O-Me-EA from the EHW Aqueous Extract

**Figure 1:**
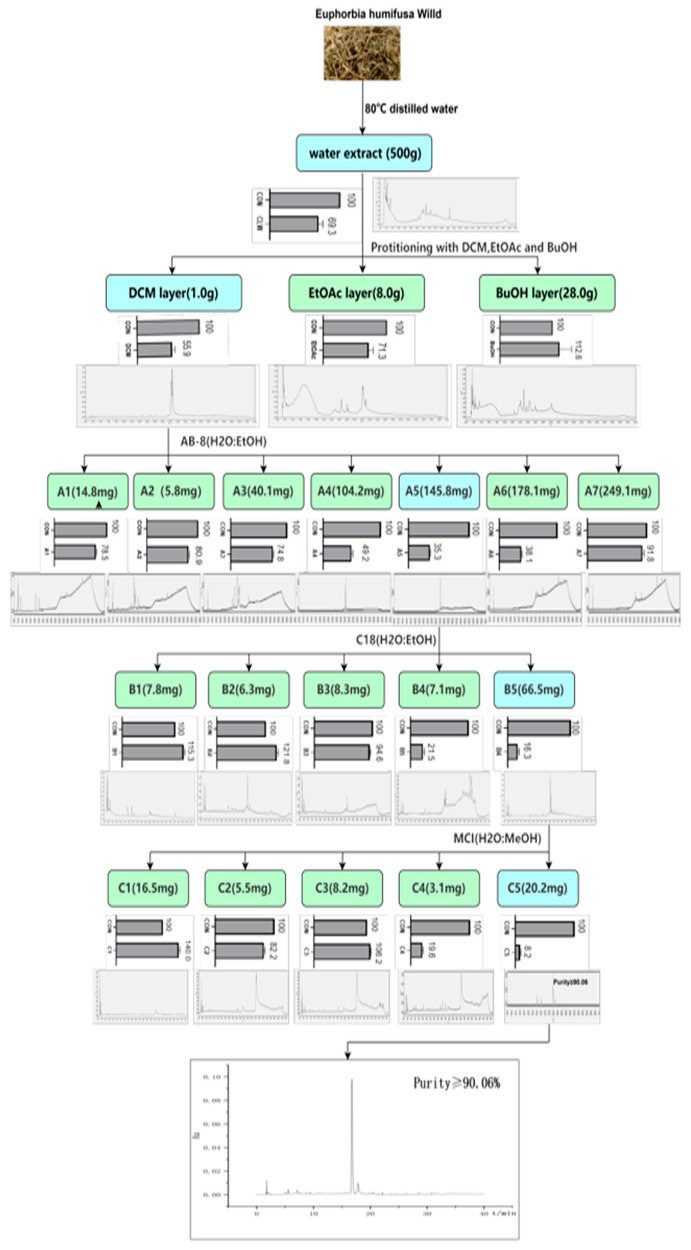
Schematic diagram of the extraction and fractions of EHW. The bar graph demonstrates the effect of the compound on the viability of 22RV-1 cells. The HPLC chromatogram illustrates the chemical fingerprints of the extract and fractions. All fractions were tested at a concentration of 0.78 μg/mL. Statistical significance was determined by one-way ANOVA, followed by Tukey’s post hoc test. All experiments were performed in triplicate (*n* = 3, mean ± SD).

EH was extracted with distilled water and yielded 600.0 g of dried EHW. The MTT results showed that EHW treatment (780 μg/mL) significantly inhibited 22RV1 cell viability. The viability rate of EHW-treated 22RV1 cells was 68.66 ± 0.40% (*p* < 0.05). The HPLC results showed that the eight peaks were contained in the fingerprint of EHW (Figure 1 and Table 1). Then, the EHW was partitioned to DCM, EtOAc, and BuOH fractions, and their 22RV1 cell viability inhibitory effects were evaluated. At the same concentration as EHW (780 μg/mL), the DCM fraction significantly suppressed the viability of 22RV1 cells, with a 50.35 ± 0.51% cell viability rate compared to the control (*p* < 0.05). The fingerprint of the DCM fraction mainly contained two peaks. In contrast, the fraction EtOAc and fraction n-BuOH showed weak or no viability inhibitory effects on the 22RV1 cells compared with the control. The viability rate of EtOAc-treated 22RV1 cells was 77.18 ± 3.54%, while the viability rate of n-BuOH-treated 22RV1 cells was 105.15 ± 5.69% (*p* < 0.05). HPLC chromatogram analysis showed that the fraction EtOAc fingerprint contained three peaks, and the fraction n-BuOH contained seven peaks (Figure 1 and Table 1). DCM fraction was further fractionated and yielded seven fractions (A1–A7). At a treatment of 780 μg/mL, these fractions exhibited varying degrees of inhibitory effects on 22RV1 cell viability. The cell viability of fractions A1–A7-treated 22RV1 cells were 99.33 ± 12.93%, 86.64 ± 1.91%, 69.17 ± 3.54%, 54.61 ± 1.91%, 37.59 ± 0.82%, 40.57 ± 1.15%, and 96.84 ± 0.99%, respectively (*p* < 0.05). Meanwhile, it was observed that fraction A1 contained two peaks in the fingerprint, fraction A2 contained three peaks in the fingerprint, fraction A3 contained five peaks in the fingerprint, fraction A4 contained two peaks in the fingerprint, fraction A5 contained one peak in the fingerprint, fraction A6 contained one peak in the fingerprint, and fraction A7 contained one peak in the fingerprint (Figure 1 and Table 1). As seen in Figure 1 and Table 1, fraction A5 exhibited the strongest inhibitory effect on the viability of 22RV1 cells. Subsequently, fraction A5 was separated into five fractions (B1–B5). Their corresponding cell viability rates in 22RV1 cells and the peaks in the HPLC fingerprints were 70.03 ± 4.95% and two peaks, 77.00 ± 9.14% and five peaks, 117.21 ± 4.32% and three peaks, 39.18 ± 2.24% and two peaks, and 24.91 ± 5.65% and one peak, respectively (*p* < 0.05). As shown in Figure 1 and Table 1, fraction B5 displayed the most potent inhibitory effect on the viability of 22RV1 cells. Therefore, fraction B5 was further purified, yielding five fractions (C1–C5). Their cell viability rates in 22RV1 cells, and the number of peaks in the fingerprints were 113.11 ± 3.41% and one peak, 93.86 ± 13.49% and three peaks, 107.55 ± 0.05% and three peaks, 30.09 ± 4.16% and seven peaks, and 11.47 ± 1.72% and three peaks, respectively (*p* < 0.05). Among them, the C5 fraction (yellow powder) exhibited the strongest effect against 22RV1 cells with a purity of ≥90.06%.

### 2.2. Identification of the 3,3′-Di-O-Me-EA

**Figure 2:**
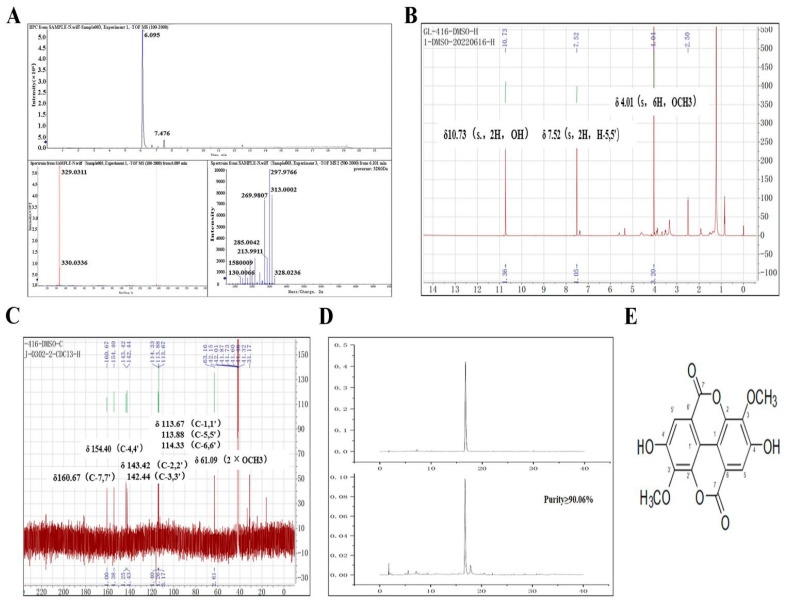
Structural analysis of 3,3′-di-O-Me-EA isolated from EHW. (**A**) HRTOF mass spectrum of 3,3′-di-O-Me-EA. (**B**) ^1^H-NMR spectrum of 3,3′-di-O-Me-EA. (**C**) ^13^C-NMR spectrum of 3,3′-di-O-Me-EA. (**D**) Comparative HPLC chromatograms of 3,3′-di-O-Me-EA and its authentic standard. (**E**) Chemical structure of 3,3′-di-O-Me-EA.

To identify the compound structure, the fraction C5 (purity ≥ 90.06%) was subjected to HRTOF mass analysis, which revealed a molecular ion peak at *m*/*z* 330.0336 [M−H] ^−^, corresponding to the molecular formula C_16_H_10_O_8_ (Figure 2A). The NMR spectroscopic data (Figure 2B,C) were acquired as follows: ^1^H NMR (400 MHz, DMSO-*d*_6_) δ: 7.52 (s, 2H, H-5, 5′), 4.01 (s, 6H, OCH_3_), and 10.73 (br. s, 2H, OH); ^13^C NMR (100 MHz, DMSO-*d*_6_) δ: 113.67 (C-1, 1′), 143.42 (C-2, 2′), 142.44 (C-3, 3′), 154.40 (C-4, 4′), 113.88 (C-5, 5′), 114.33 (C-6, 6′), 160.67 (C-7, 7′), and 61.09 (2 × OCH_3_). By comparing the ^1^H (Figure 2C) and ^13^C (Figure 2D) NMR data with those reported in the previous literature [39,40], the compound was identified as 3,3′-di-O-Me-EA. This identification was further confirmed by HPLC co-injection of fraction C5 and 3,3′-di-O-Me-EA standards (Figure 2D) with identical retention times.

### 2.3. In Silico ADMET Analysis

In silico ADMET prediction was regarded as an effective tool for drug design with less costly and time-saving properties. Hence, the drug probability and pharmacokinetic properties of 3,3′-di-O-Me-EA were evaluated. The data listed in Table 2 show that 3,3′-di-O-Me-EA contains two rotatable bonds, eight hydrogen bond acceptors, and two hydrogen bond donors, which together contribute to a total polar surface area (TPSA) of 119.34 Å. The logP value (1.79) indicated that 3,3′-di-O-Me-EA has moderate lipophilicity and a favorable molecular size for membrane permeability and bioavailability. The drug-likeness evaluation results showed that 3,3′-di-O-Me-EA complies with multiple established rules, including Lipinski, Ghose, Veber, Egan, and Muegge filters. Moreover, we also predicted the pharmacokinetic properties such as absorption, distribution, metabolism, excretion, and toxicity profiles of 3,3′-di-O-Me-EA. The results demonstrated that 3,3-di-O-Me-EA has moderate water solubility (logS = −3.01) and high predicted human intestinal absorption (82.6%), indicating efficient oral uptake. Its low Caco-2 permeability (log Papp = −5.75) suggests limited passive diffusion. Importantly, it is neither a substrate nor an inhibitor of P-gp, implying low risk of efflux-mediated bioavailability reduction. It also has a low VDss (−0.69 log L/kg) and high plasma protein binding (82.4%), indicating limited tissue distribution. Its low BBB permeability (6.4%) suggests minimal CNS exposure. For metabolism, it is not a substrate of major CYP enzymes but inhibits CYP1A2 (82%) and CYP2C9 (56.2%), indicating potential drug–drug interaction risk. It is predicted to be a UGT substrate (84.8%). It exhibits low total clearance (35%) and is not an OCT2 substrate, suggesting stable elimination. Toxicity assessment shows no hERG inhibition (9.1%), reflecting favorable cardiovascular safety. Environmental toxicity predictions (T. pyriformis) also indicate low ecological risk. However, the positive AMES test indicates a potential mutagenic risk, warranting experimental validation. In short, 3,3′-di-O-Me-EA exhibited favorable drug-like and pharmacokinetic properties.

### 2.4. 3,3′-Di-O-Me-EA Interaction with VDAC1

**Figure 3:**
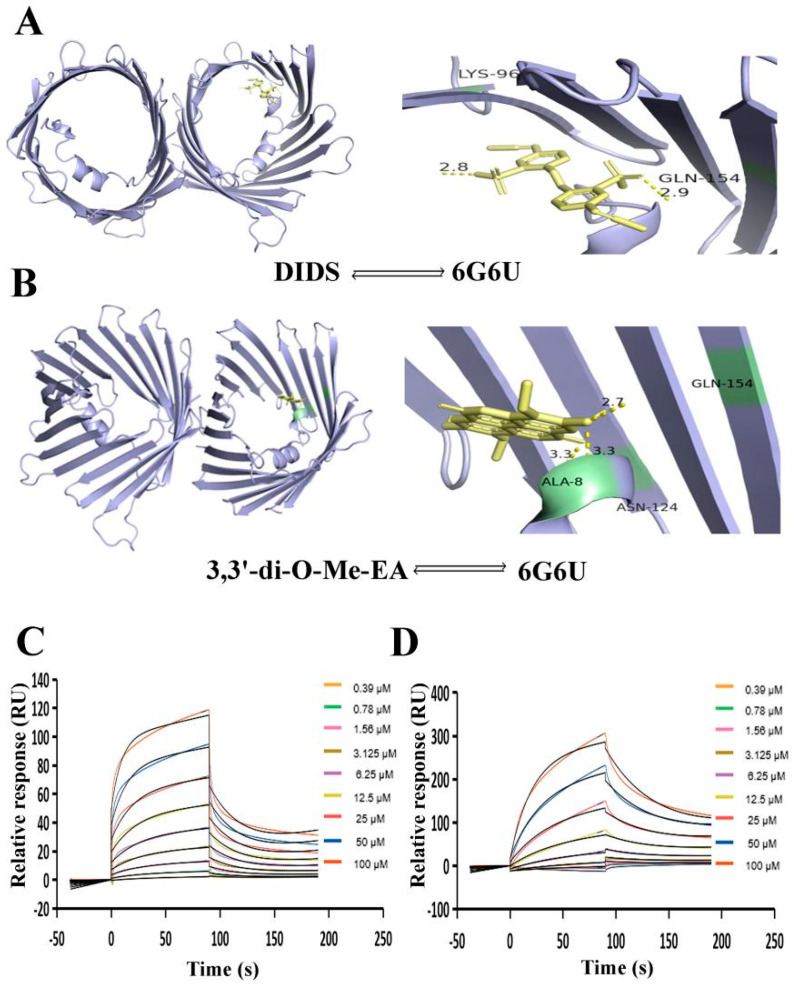
Docking and SPR analysis of 3,3′-di-O-Me-EA and the inhibitor DIDS. (**A**) Binding pose of DIDS within VDAC1 (PDB: 2AX6). Interaction of DIDS with VDAC1 amino acid residues. The yellow dashed lines indicate hydrogen bonds. (**B**) Binding pose of 3,3′-di-O-Me-EA within VDAC1 (PDB: 6G6U). Interaction of 3,3′-di-O-Me-EA with VDAC1 amino acid residues. The yellow dashed lines indicate hydrogen bonds. (**C**) Interaction between 3,3′-di-O-Me-EA and DIDS as determined by SPR. The two lines of each color denote the experimental results and their corresponding fitted curves. (**D**) Interaction between 3,3′-di-O-Me-EA and VDAC1 as determined by SPR. The two lines in each color denote the experimental results and their corresponding fitted curves.

Molecular docking studies were performed to predict the potential binding affinity of 3,3′-di-O-Me-EA and the VDAC1 protein. The docking results showed that the VDAC1 protein demonstrated a higher binding affinity for 3,3′-di-O-Me-EA (Table 3, Figure 3A,B). The binding score of VDAC1 and 3,3′-di-O-Me-EA (−7.5 kcal/mol) was higher than the binding score of VDAC1 with its inhibitor DIDS (−6.8 kcal/mol). As shown in Figure 3A,B and Table 3, 3,3′-di-O-Me-EA was closely related to VDAC1, with three hydrogen bonds formed with residues GLN-154, ALA-8, and ASN-124 (Table 3). Moreover, to confirm the molecular docking results, SPR analysis was conducted. The results demonstrated that (Figure 3C,D) 3,3′-di-O-Me-EA and DIDS could bind to the VDAC1 protein in a concentration-dependent manner. Their Kd was 46.0 µM and 22.6 μM, respectively. These results indicated that 3,3′-di-O-Me-EA may bind to the VDAC1 protein.

### 2.5. 3,3′-Di-O-Me-EA Suppresses PCa Cell Viability and Induces Apoptosis

**Figure 4:**
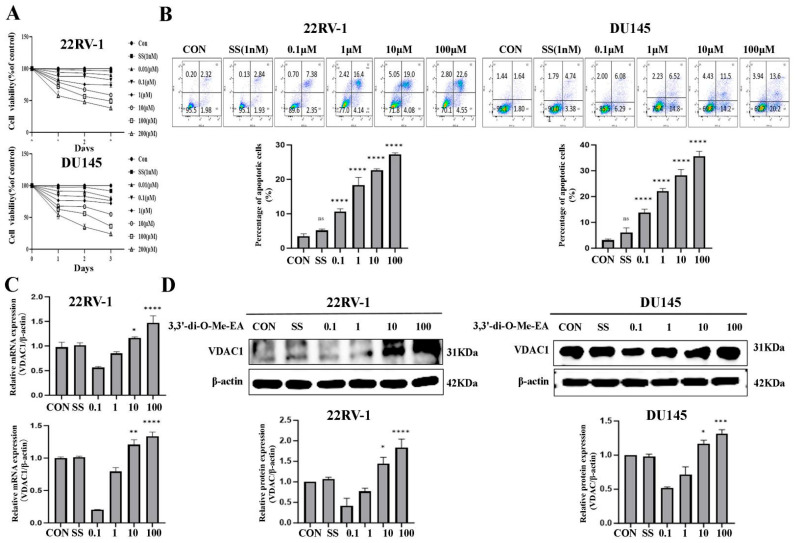
Effects of 3,3′-di-O-Me-EA on the cell viability, apoptosis, and VDAC1 gene/protein expression in 22RV-1 and DU145 cells. (**A**) Cell viability of 3,3′-di-O-Me-EA (0.1–100 μM) and SS (1 nM)-treated 22RV-1 and DU145 cells for 24, 48, and 72 h. (**B**) Apoptosis of 3,3′-di-O-Me-EA (0.1–100 μM) and SS (1 nM)-treated 22RV-1 and DU145 cells for 24 h. (**C**) Dose effects of 3,3′-di-O-Me-EA on the VDAC1 mRNA expression levels. The 22RV-1 and DU145 cells were treated with 3,3′-di-O-Me-EA (0.1–100 μM) and SS (1 nM) for 24 h. (**D**) Dose effects of 3,3′-di-O-Me-EA on the VDAC1 protein expression levels. The 22RV-1 and DU145 cells were treated with 3,3′-di-O-Me-EA (0.1–100 μM) and SS (1 nM) for 24 h. Statistical significance was determined by one-way ANOVA, followed by Tukey’s post hoc test. All experiments were performed in triplicate (*n* = 3, mean ± SD). Statistical significance was set at ns *p* > 0.05; * *p* < 0.05, ** *p* < 0.01, *** *p* < 0.001 and **** *p* < 0.0001.

The viability inhibitory effects of 3,3′-di-O-Me-EA in 22RV-1 and DU145 PCa cells were assessed using the MTT assay. The results demonstrated that the viability of both 22RV-1 and DU145 cells was significantly decreased in a time- and dose-dependent manner upon treatment with 3,3′-di-O-Me-EA, whereas SS treatment (1 nM) did not inhibit the viability of either 22RV-1 or DU145 cells (Figure 4A) (*p* < 0.05). Flow cytometric analysis revealed that 3,3′-di-O-Me-EA dose-dependently induced apoptosis in both 22RV-1 and DU145 cells. However, SS treatment (1 nM) cannot alter the apoptosis of 22RV-1 and DU145 cells (Figure 4B) (*p* < 0.05). Collectively, these findings suggest that 3,3′-di-O-Me-EA may inhibit PCa cells via inducing apoptosis.

### 2.6. 3,3′-Di-O-Me-EA Up-Regulates VDAC1 mRNA and Protein Expression in PCa Cells

We previously demonstrated that EHW up-regulated the VDAC1 protein expression level in both 22RV1 and DU145 cells. Thus, the VDAC1 mRNA and protein expression regulatory effects of 3,3′-di-O-Me-EA in 22RV1 and DU145 cells were evaluated. q-PCR results indicated that treatment with different concentrations of 3,3′-di-O-Me-EA led to a dose-dependent up-regulation of the VDAC1 mRNA expression level in both cell lines, but SS cannot alter the VDAC1 mRNA expression level in both 22RV1 and DU145 cells (Figure 4C) (*p* < 0.05). Consistently, Western blot analysis further demonstrated that 3,3′-di-O-Me-EA treatment resulted in a dose-dependent increase in the VDAC1 protein expression level in PCa (22RV1 and DU145) cells (Figure 4D) (*p* < 0.05). These results suggest that VDAC1 may play an important role in the anti-PCa effect of 3,3′-di-O-Me-EA.

### 2.7. VDAC1 Depletion Suppresses PCa Cell Viability Inhibitory and Apoptosis Induction Effects of 3,3′-Di-O-Me-EA

**Figure 5:**
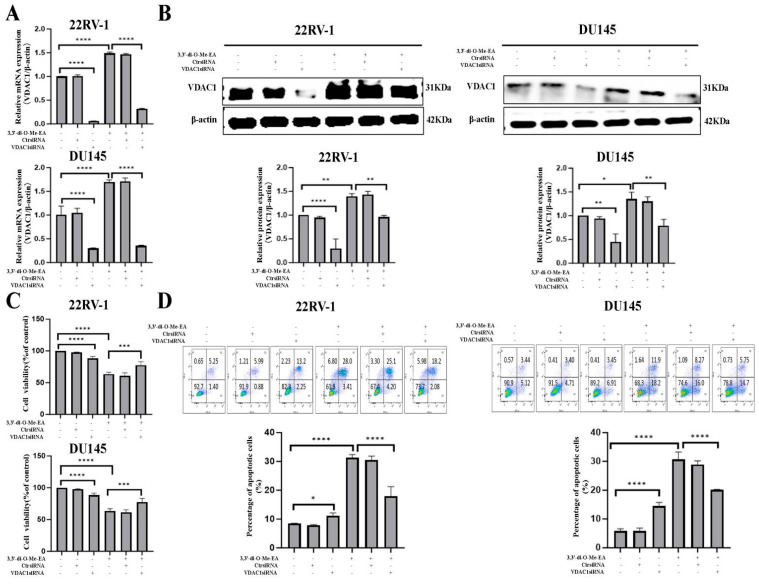
Effects of 3,3′-di-O-Me-EA on the cell viability, apoptosis, and VDAC1 gene/protein expression in VDAC1-depleted 22RV-1 and DU145 cells. (**A**) VDAC1 mRNA expressions in VDAC1-depleted 22RV-1 and DU145 cells with or without 3,3′-di-O-Me-EA (100 μM) treatment for 24 h. (**B**) VDAC1 protein expression in VDAC1-depleted 22RV-1 and DU145 cells with or without 3,3′-di-O-Me-EA (100 μM) treatment for 24 h. (**C**) Cell viability of VDAC1-depleted 22RV-1 and DU145 cells with or without 3,3′-di-O-Me-EA (100 μM) treatment for 24 h. (**D**) Apoptosis of VDAC1-depleted 22RV-1 and DU145 cells with or without 3,3′-di-O-Me-EA (100 μM) treatment for 24 h. Statistical significance was determined by one-way ANOVA, followed by Tukey’s post hoc test. All experiments were performed in triplicate (*n* = 3, mean ± SD). Statistical significance was set at * *p* < 0.05, ** *p* < 0.01, *** *p* < 0.001 and **** *p* < 0.0001.

To determine whether 3,3′-di-O-Me-EA inhibits PCa cells and induces apoptosis via regulating VDAC1, VDAC1siRNA-transfected 22RV-1 and DU145 cells were used. qPCR and Western blot results showed that the VDAC1 mRNA and protein expression levels were significantly down-regulated in the VDAC1-depleted 22RV-1 and DU145 cells (Figure 5A,B). Moreover, compared with the 22RV-1 and DU145 cells, 3,3′-di-O-Me-EA treatment significantly down-regulated VDAC1 mRNA and protein expression levels in VDAC1-depleted 22RV-1 and DU145 cells (*p* < 0.05). Furthermore, the results showed that the viability of the VDAC1-depleted 22RV-1 and DU145 cells was decreased, and apoptosis was increased to some extent compared with the control (Figure 5C,D). However, compared with the 22RV-1 and DU145 cells, 3,3′-di-O-Me-EA treatment significantly increased cell viability and decreased apoptosis of VDAC1-depleted 22RV-1 and DU145 cells, suggesting that VDAC1 depletion may suppress the anti-PCa effects of 3,3′-di-O-Me-EA (*p* < 0.05).

### 2.8. VDAC1 Overexpression Enhances PCa Cell Viability Inhibitory and Apoptosis Induction Effects of 3,3′-Di-O-Me-EA

**Figure 6:**
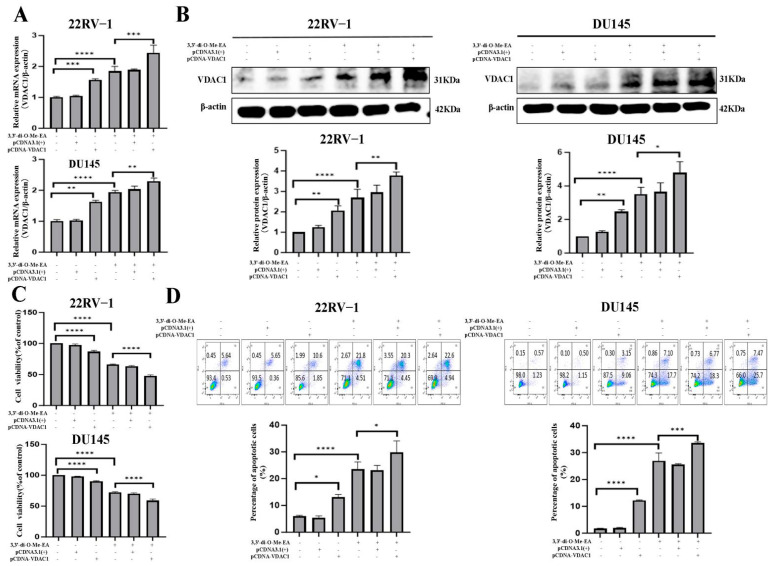
Effects of 3,3′-di-O-Me-EA on the cell viability, apoptosis, and VDAC1 gene/protein expression in VDAC1-overexpressed 22RV-1 and DU145 cells. (**A**) VDAC1 mRNA expressions in VDAC1-overexpressed 22RV-1 and DU145 cells with or without 3,3′-di-O-Me-EA (100 μM) treatment for 24 h. (**B**) VDAC1 protein expression in VDAC1-overexpressed 22RV-1 and DU145 cells with or without 3,3′-di-O-Me-EA (100 μM) treatment for 24 h. (**C**) Cell viability of VDAC1-overexpressed 22RV-1 and DU145 cells with or without 3,3′-di-O-Me-EA (100 μM) treatment for 24 h. (**D**) Apoptosis of VDAC1-overexpressed 22RV-1 and DU145 cells with or without 3,3′-di-O-Me-EA (100 μM) treatment for 24 h. Statistical significance was determined by one-way ANOVA, followed by Tukey’s post hoc test. All experiments were performed in triplicate (*n* = 3, mean ± SD). Statistical significance was set at * *p* < 0.05, ** *p* < 0.01, *** *p* < 0.001 and **** *p* < 0.0001.

Next, the cell viability and apoptosis regulatory effects of 3,3′-di-O-Me-EA in VDAC1-overexpressed 22RV-1 and DU145 cells were studied using the pCDNA-VDAC1 plasmid. It was observed that the VDAC1 mRNA and protein expression levels were significantly upregulated in both VDAC1-overexpressed 22RV-1 and DU145 cells compared with the control (Figure 6A,B). Of note, compared with the 22RV-1 and DU145 cells, 3,3′-di-O-Me-EA treatment significantly upregulated VDAC1 mRNA and protein expression levels in VDAC1-overexpressed 22RV-1 and DU145 cells (*p* < 0.05). Moreover, the results showed that the viability of the VDAC1-overexpressed 22RV-1 and DU145 cells was decreased, and apoptosis was increased to some extent compared with the control (Figure 6C,D). Furthermore, compared with the 22RV-1 and DU145 cells, 3,3′-di-O-Me-EA treatment significantly decreased cell viability and increased apoptosis of VDAC1-overexpressed 22RV-1 and DU145 cells, suggesting that VDAC1 overexpression may enhance anti-PCa effects of 3,3′-di-O-Me-EA (*p* < 0.05).

## 3. Discussion

PCa is a common malignant tumor with a high morbidity and mortality rate in men globally [41]. Despite various therapies used in the treatment of PCa, with some merits, there is still an urgent need to search for new drugs with a clear mechanism of action [42]. It has been reported that some species of the genus Euphorbia have potent anti-tumor effects in various types of cancer. For example, Euphorbia Pekinensis extract significantly inhibits CRC cell growth in vitro. Moreover, it synergizes with PD-1 blockade to inhibit tumorigenesis in vivo [43]. Euphorbia helioscopia granules concentration-dependently inhibit the proliferation, invasion, and migration of PC-9 and A549 cells, significantly induce cell apoptosis, up-regulate E-cadherin and Bax protein expressions, and down-regulate Bcl-2, vimentin, MMP2, and MMP9 protein expressions [44]. In addition, Euphorbia fischeriana-diterpenoids strongly inhibited C4-2B and C4-2B/ENZR PCa cell viability [45,46]. Our previous article also documented that EHW suppressed 22RV-1 cell viability in a concentration-dependent manner, and some of its active compounds down-regulated AR protein expression [38]. However, the anti-PCa effects and molecular mechanisms of active compounds derived from EH need to be elucidated.

In this study, the 3,3′-di-O-Me-EA was isolated from EH using bioassay-guided fractionation, and its structure was identified by HRTOF mass, NMR, and an authentic standard. 3,3′-di-O-Me-EA was then observed with favorable physicochemical properties, oral bioavailability, moderate tissue distribution, low metabolic liability, and low toxicity profile. However, potential drug–drug interaction risk and mutagenicity need further experimental validation. Pharmacological studies have demonstrated that 3,3′-di-O-Me-EA inhibits M1 macrophage polarization and COX-2 secretion, reduces lipid accumulation in adipocytes, and stabilizes atherosclerotic plaques via PPARγ regulation [47]. It has been reported that 3,3′-di-O-Me-EA significantly inhibits PTP1B activity. PTP1B is an important regulator in insulin signaling and plays a key role in some metabolic diseases such as type 2 diabetes [48]. 3,3′-di-O-Me-EA also regulates inflammatory and metabolic disorders [49]. Even so, no article has documented its anti-cancer effects. Our MTT and flow cytometric analysis results demonstrated for the first time that 3,3′-di-O-Me-EA significantly inhibited 22RV-1 and DU145 cell viability and induced apoptosis in both PCa cell lines. Of note, 3,3′-di-O-Me-EA showed better cell viability inhibitory and apoptosis induction effects in DU145 cells than in 22RV-1 cells, suggesting that 3,3′-di-O-Me-EA may be more sensitive to androgen-insensitive PCa cell lines. However, SS did not affect the cell viability or apoptosis of 22RV1 and DU145 cells.

We recently observed that EHW up-regulated the VDAC1 protein expression in both 22RV1 and DU145 cells. VDAC1 is the most abundant protein in the mitochondrial outer membrane [50]; it belongs to the VDAC protein family [51] and exhibits dual or context-dependent regulatory roles in cancer development [52]. It has been reported that VDAC1 plays a critical role in mitochondria-dependent apoptosis [53,54]. VDAC1 oligomerization or the opening of the mitochondrial permeability transition pore (mPTP) induces the release of cytochrome c and other death factors into the cytosol, thereby activating caspase cascades and inducing apoptosis. VDAC1 also prevents cancer cell apoptosis by binding to hexokinase II (HK-II) or anti-apoptotic Bcl-2 family proteins like Bcl-xL [55]. VDAC1 down-regulation promotes metabolic rewiring, suppresses tumor growth, induces apoptosis, and modulates the tumor micro-environment [56,57]. Overexpression of VDAC1 induces its transition from monomers to oligomers, which subsequently triggers cell death [55]. It has been reported that some anti-cancer agents suppress PCa cell proliferation via regulating VDAC1 activity. For example, cannabidiol induces apoptosis in PCa cells by modulating the interaction between VDAC1 and HK-II [58]. Another study indicated that circ-0001326 regulates PCa cell proliferation partly through mediating the miR-577/VDAC1 axis [59]. However, the role of VDAC1 in PCa progression remains unclear [60]. Therefore, in this study, the regulatory effect of the VDAC1 protein on the action of 3,3′-di-O-Me-EA against PCa cells was further studied. The molecular docking results predicted that 3,3′-di-O-Me-EA interacts with the VDAC1 protein. The SPR results further confirmed that 3,3′-di-O-Me-EA may bind to the VDAC1 protein, with a Kd value of (46.0 µM). Moreover, q-PCR and Western blot results demonstrated that 3,3′-di-O-Me-EA up-regulated VDAC1 mRNA and protein expression levels in 22RV1 and DU145 cells in a dose-dependent manner. However, inconsistent results were observed compared with the previous article reported by P. Sáez-Martínez et al. [61] that SS treatment did not alter either VDAC1 mRNA or protein levels in 22RV1 and DU145 cells, indicating that the up-regulation of the VDAC1 protein expression by SS in PCa may be cell-type-dependent. Consequently, after knockdown of VDAC1 in 2RV-1 and DU145 cells, 3,3′-di-O-Me-EA treatment significantly down-regulated VDAC1 mRNA and protein expression levels, increased cell viability, and decreased apoptosis. Moreover, 3,3′-di-O-Me-EA treatment significantly up-regulated VDAC1 mRNA and protein expression levels, inhibited cell viability, and induced apoptosis in VDAC1-overexpressed 22RV-1 and DU145 cells. These results suggested that 3,3′-di-O-Me-EA inhibited 22RV-1 and DU145 cell viability and induced apoptosis via up-regulation of VDAC1 gene and protein expression levels. Our results also demonstrated that VDAC1 depletion or VDAC1 overexpression inhibited 22RV-1 and DU145 cell viability and induced apoptosis to some extent. It is not surprising that some articles have documented the cell proliferation inhibitory and apoptosis induction effects of both VDAC1-depleted or VDAC1-overexpressed cancer cells, including glioblastoma [62], bladder cancer [63], uterine cancer [64], mesothelioma [56], lung cancer, PCa, pancreas cancer, colon carcinoma and hepatocellular carcinoma [65], cervical cancer [66,67], and cervix squamous cell carcinoma [68].

In short, we first demonstrated the anti-PCa activity and VDAC1-mediated mechanisms of 3,3′-di-O-Me-EA derived from EH. However, our study has certain limitations. For example, all the experiments were conducted using in vitro assays; animal models, including transgenic animals, are needed to confirm the results. Moreover, we demonstrated that VDAC1 mediated the anti-PCa activity of 3,3′-di-O-Me-EA. But the mechanistic regulatory effect of VDAC1 on the PCa progression and related signal pathways must be elucidated. In addition, we previously demonstrated that EHW and quercetin, kaempferol, and luteolin down-regulated AR protein expression [38]. But the interaction of VDAC1 and AR and the regulatory effect of VDAC1 and AR interaction on the action of 3,3′-di-O-Me-EA against PCa are not clear yet and must be further clarified.

## 4. Materials and Methods

### 4.1. Materials

HPLC-grade methanol, acetonitrile, ultrapure water, and 0.05% formic acid were obtained from TEDIA Company, Inc. (Fairfield, OH, USA). An Acquity^TM^ ultra high-performance liquid chromatograph (Waters, Milford, MA, USA) and a Triple TOF 6600+ time-of-flight mass spectrometer with electrospray ionization source (AB SCIEX, Framingham, MA, USA) were used. The human prostate cancer 22RV1 cells were obtained from Shanghai Mingjing Biotechnology Co., Ltd. (Shanghai, China). Fetal bovine serum (FBS), MTT, penicillin, and streptomycin were purchased from Invitrogen (Grand Island, NY, USA). Trypsin-EDTA (0.25%) was acquired from Life Technologies (New York, NY, USA). 3,3′-Di-O-methylellagic acid was supplied by Yunnan Xili Biotechnology Co., Ltd. (Kunming, China). The Apoptosis Assay Kit was procured from Service Bio-Biotechnology Co., Ltd. (Wuhan, China). Antibodies against VDAC1, as well as goat anti-mouse IgG and goat anti-rabbit IgG, were obtained from Abcam (Cambridge, MA, USA). The β-actin antibody was purchased from Sigma-Aldrich (St. Louis, MO, USA). The BCA protein quantification kit was obtained from Solar Bio-Science & Technology Co., Ltd. (Beijing, China). RIPA lysis buffer was acquired from Epizyme Biomedical Technology Co., Ltd. (Shanghai, China). The Rapid Preparation Kit for 10% SDS-PAGE gels was provided by Beijing Biotechnic Biotechnology Co., Ltd. (Beijing, China). ECL chemiluminescence substrate was procured from Bio sharp Life Sciences (Beijing, China). The 20× TBST buffer solution was supplied by Solar-bio (Beijing, China). The RT-qPCR kit was purchased from Takara Biomedical Technology Co., Ltd. (Dalian, China).

### 4.2. Plant Material Collection and Processing

The EH plant material was purchased from Xinjiang Hetian Madisen Co., Ltd. (Hetian, China). A voucher specimen (Voucher No. C30003513) was authenticated by Dr. Aikebaier Maimaiti in the lab center of the school of pharmacy of Xinjiang Medical University. The EH samples were cleaned and air-dried in the shade. Then, the samples were ground and sieved through a 20-mesh sieve, and the powder was stored at −20 °C until use.

### 4.3. Extract Preparation

The dried EH (20 kg) powder was extracted with distilled water (80 °C) at a solid-to-liquid ratio of 1:10 (*w*/*v*) for 60 min. Then, it was concentrated under reduced pressure and subsequently yielded a crude extract (600.0 g). Thereafter, 500.0 g of the crude extract was fractionated using dichloromethane (DCM), ethyl acetate (EtOAc), and n-butanol (n-BuOH), producing the DCM (1.0 g), EtOAc (8.0 g), and n-BuOH (28.0 g) fractions, respectively. The active fraction DCM was subjected to an AB-8 microporous resin column with eluting aqueous ethanol gradient system (0–95%) and yielded A1 (14.8 mg), A2 (5.8 mg), A3 (40.1 mg), A4 (104.2 mg), A5 (145.8 mg), A6 (178.1 mg), and A7 (249.1 mg) fractions. The active fraction A5 was further fractionated using a reversed-phase C18 column (eluted with 10–95% aqueous ethanol) and produced B1 (7.8 mg), B2 (6.3 mg), B3 (8.3 mg), B4 (7.1 mg), and B5 (66.5 mg) fractions. The most active fraction B5 was subjected to an MCI gel column with a gradient of aqueous ethanol (10–95%) to yield C1 (16.5 mg), C2 (5.5 mg), C3 (8.2 mg), C4 (10.1 mg), and C5 (13.2 mg) fractions. Then, the chemical structure of active fraction C5 was identified using HRTOF mass, NMR, and a standard. The bioactivity of the extract and all fractions was screened using the MTT assay, and chemical fingerprints were obtained using the high-performance liquid chromatography (HPLC) method.

### 4.4. HPLC Analysis of EHW and Fractions

HPLC analysis was performed using a YMC-C18 column (5 µm, 4.6 mm × 250 mm). The mobile phase consisted of 0.1% formic acid in water (A) and 0.1% formic acid in acetonitrile (B). A linear gradient elution was applied as follows: 0 min, 5% B; 20 min, 16% B; 30 min, 20% B; 42 min, 25% B; 50 min, 48% B; 60 min, 82% B; and 65 min, 5% B. The flow rate was 1.0 mL/min, the column temperature was maintained at 25 °C, and the detection wavelength was set at 254 nm. The injection volume was 5 µL.

### 4.5. UPLC-Triple-TOF/MS Condition

Chromatographic separation was performed on a Waters UPLC system (Waters Corp., Milford, MA, USA). An ACQUITY UPLC T3 column (1.8 µm, 2.1 × 150 mm; Waters Corp.) was used in all the chromatographic experiments. The mobile phases were 0.1% formic acid–water (A) and 0.1% formic acid–acetonitrile (B). The linear gradient programs were as follows: 0 min, 5% B; 14 min, 95% B; and 16 min, 95% B. The sample injection volume was 3 µL, the column oven temperature was maintained at 45 °C, and the flow rate was set at 0.3 mL/min.

Mass spectrometric analysis was conducted using an AB Triple TOF 6600plus System (Framingham, MA, USA). The optimal MS conditions were as follows: In positive ion mode, the source voltage was +5.5 kV, and the source temperature was 550 °C. In negative ion mode, the source voltage was −4.5 kV, and the source temperature was 550 °C. The pressures of Gas 1 (Air) and Gas 2 (Air) were set to 55 psi. The pressure of Curtain Gas (N_2_) was set to 35 psi. The maximum allowed error was set to ±5 ppm. Declustering potential (DP) was set at 80 V, and collision energy (CE) was set at 10 V. For MS/MS acquisition mode, the parameters were almost the same, except that the collision energy (CE) was set at ±40 ±20 V, ion release delay (IRD) at 67, and ion release width (IRW) at 25. The IDA-based auto-MS^2^ was performed on the 8 most intense metabolite ions in a cycle of full scan (1 s). The scan ranges of *m*/*z* for precursor ion and product ion were set as 100–1500 Da and 50–1500 Da, respectively. The exact mass calibration was performed automatically before each analysis employing the Automated Calibration Delivery System.

### 4.6. NMR Analysis

The high-purity sample was accurately weighed and dissolved in deuterated dimethyl sulfoxide (DMSO-*d*_6_) for analysis. All NMR experiments were conducted at 298 K. The chemical shifts (δ) for ^1^H and ^13^C NMR spectra were referenced to tetramethyl silane (TMS) as an internal standard, and the coupling constants (J) were reported in Hertz (Hz).

### 4.7. In Silico ADME Screening

The ADME screening of 3,3′-di-O-Me-EA was carried out on the Swiss ADME online platform (http://www.swissadme.ch/index.php accessed on 13 November 2025) using Lipinski Rule of five and Ghose, Veber, Egan, and Muegge to systematically evaluate the drug-likeness of the 3,3′-di-O-Me-EA. The toxicity screening of 3,3′-di-O-Me-EA was conducted using the admetSAR 3.0 platform.

### 4.8. Molecular Docking

The three-dimensional structures of the small molecule ligands were retrieved from the PubChem database using their compound IDs (CID: 5488919 for 3,3′-di-O-Me-EA and CID: 50600 for DIDS). The crystal structure of the target protein VDAC1 was obtained from the Protein Data Bank (PDB) under accession code 6G6U in PDB format. Molecular docking was performed using the CB-Dock 2 online server (https://cadd.labshare.cn/cb-dock2/ accessed on 23 October 2023). CB-Dock 2 automatically identifies ligand-binding cavities and generates the docking grid box without requiring manual input. Both protein and ligand structures were submitted as inputs to predict the binding poses and affinities using the Auto Dock Vina scoring function. The results of docking complexes were visualized and analyzed using the PyMOL molecular graphics system (version 2.5).

### 4.9. SPR Analysis

VDAC1 protein was immobilized on a CM5 sensor chip to a level of 5500 response units (RUs) using Bia core (8K) according to the manufacturer’s protocol. For steady-state affinity analysis, the 3,3′-di-O-Me-EA and DIDS were separately dissolved in 1 × PBS-P+ buffer containing 5% DMSO at concentrations of 0.39, 0.78, 1.56, 3.125, 6.25, 12.5, 25, 50, and 100 μM, and were run across the chip. Each sample that was bound to the surface was associated for 90 s at a flow rate of 30 μL/min. Dissociation of sensor chips was performed for 90 s. Due to the refractive properties of DMSO, four running buffers with different DMSO concentrations (1 × PBS-P+ containing 4.2%, 4.6%, 5.4%, and 5.8% DMSO) were additionally prepared for solvent correction at the beginning and end of the running process. The dissociation constant (KD) was fitted and recorded by Biacore Insight Evaluation Software 5.0.18.22102 (Cytiva) using the One-to-One analysis model.

### 4.10. Cell Culture

The 22RV-1 cells were cultured in H1650 medium, and DU145 cells were cultured in DU145 cell special medium with 10% fetal bovine serum and 1% penicillin/streptomycin (Hyclone, Hyde Park, UT, USA). The cultured cells were maintained at 37 °C in a humidified incubator with 5% CO_2_.

### 4.11. Cytotoxicity (MTT) Assay

Cells were seeded in 96-well plates at a density of 6 × 10^4^ cells/mL and incubated for 24 h. The medium was then replaced, and the cells were cultured with serum-containing medium (Gibco, Waltham, MA, USA) with different concentrations (0, 0.1, 1, 10, and 100 µM) of 3,3′-di-O-Me-EA and SS (1 nM) for 24, 48, and 72 h. At each time point, 5 mg/mL of MTT solution was added, and the cells were incubated at 37 °C for 4 h. Subsequently, 150 µL of DMSO was added to each well to dissolve the formazan crystals. The optical density (OD) was measured at a wavelength of 570 nm using a microplate reader.

### 4.12. Cell Apoptosis Assay

The 22RV-1 and DU145 cells were seeded in 6-well plates at a concentration of 2.0 × 10^6^ cells/well. When the cell density reached over 90%, they were treated with different concentrations (0, 0.1, 1, 10, and 100 µM) of 3,3′-di-O-Me-EA and SS (1 nM) for 24 h. Then, the cell was resuspended in 100 µL of 1× Annexin V binding buffer and stained with 5 µL of annexin V-FITC and 5 µL of propidium iodide (PI). After incubation in the dark at room temperature for 15 min, 400 µL of 1× Annexin V binding buffer was added to each tube. Apoptosis was detected using a BD LSRII flow cytometer.

### 4.13. RT-qPCR Analysis

The 22RV-1 and DU145 cells were seeded in 6-well plates at a density of 2.0 × 10^6^ cells/well. When the cell confluence reached over 90%, they were treated with various concentrations (0, 0.1, 1, 10, and 100 μM) of 3,3′-di-O-Me-EA, along with the SS (1 nM), for 24 h. Then, the cells were collected by centrifugation at 1000 rpm and 4 °C for 10 min. The supernatant was carefully discarded, and the cell pellets were washed twice with 1 mL of PBS. Total RNA was isolated from the cell pellets using Trizell reagent, and 1 μg of it was reverse-transcribed to cDNA using the 5× Prime Script RT Premix. Then, template cDNA was amplified using a 2× Cham Q Universal SYBR qPCR Master Mix (Vazyme Biotech, Nanjing, China), according to the manufacturer’s procedure. The qPCR reactions were carried out in a Quant Studio 5 Real-Time PCR System. The primer sequences used for PCR amplification are listed in Supplementary Table S1. β-actin was used as an internal reference, and the relative expression of mRNA was obtained using the 2^−ΔΔCT^ method.

### 4.14. WB Analysis

The 22RV-1 and DU145 cells were seeded in 6-well plates at a density of 2.0 × 10^6^ cells/well. Upon reaching over 90% confluence, the cells were treated with varying concentrations (0, 0.1, 1, 10, and 100 μM) of 3,3′-di-O-Me-EA and SS (1 nM) for 24 h. Then, the cells were harvested by centrifugation at 1000× *g* for 10 min at 4 °C and lysed on ice using RIPA buffer for 20 min. The lysates were centrifuged at 12,000× *g* for 20 min at 4 °C, and the supernatants were collected for protein quantification using the BCA assay. Protein samples were mixed with 5× loading buffer, denatured by heating at 100 °C for 10 min, and cooled on ice. An equal amount (15 μg) of total protein/lane was separated by electrophoresis on the 10% SDS-polyacrylamide gel electrophoresis and then transferred onto a PVDF membrane. The membranes were blocked with 5% non-fat milk for 1 h at room temperature, and then incubated overnight at 4 °C with antibody against VDAC1 (1:1000), and β-actin antibody (1:10,000), followed by incubation with goat anti-mouse IgG (1:10,000) and goat anti-rabbit IgG (1:10,000) secondary antibodies for 1 h at room temperature. Protein bands were visualized using enhanced chemiluminescence (ECL) detection.

### 4.15. siRNA-VDAC1 and Cell Transfection

When the 22RV1 and DU145 cells reached 30–35% confluence in antibiotic-free medium, the siRNA-VDAC1 transfection was conducted. VDAC1 siRNA or control siRNA (0.5 µL, 10 µM, 1 pmol each) was diluted in 25 µL of Opti-MEM™ reduced-serum medium to a final concentration of 50 nM. Lipofectamine™ 2000 (0.5 µL) was diluted in 25 µL of Opti-MEM™ medium and incubated at room temperature for 5 min. The diluted siRNA and diluted Lipofectamine™ 2000 were gently mixed and incubated at room temperature for 20 min to form transfection complexes, according to the manufacturer‘s protocol. The mixture was added to cells, and the cells were further cultured for 24 h at 37 °C in a humidified incubator with 5% CO_2_. Then, the siRNA-VDAC1-transfected 22RV1 and DU145 cells were used for subsequent analyses, including the MTT assay, apoptosis detection, Western blot, and PCR.

### 4.16. pCDNA-VDAC1 and Cell Transfection

The pCDNA-VDAC1 transfection was carried out when 22RV1 and DU145 cells reached 70–80% confluence in antibiotic-free medium. The pCDNA-VDAC1 or pCDNA3.1(+) vector (0.1 µL each) was diluted in 25 µL of Opti-MEM™ reduced-serum medium. Lipofectamine™ 2000 (0.1 µL) was diluted in 25 µL of Opti-MEM™ medium and incubated at room temperature for 5 min. The diluted plasmid DNA and diluted Lipofectamine™ 2000 were gently mixed and incubated at room temperature for 20 min to form transfection complexes, according to the manufacturer‘s protocol. The mixture was added to cells, and the cells were further cultured for 24 h at 37 °C in a humidified incubator with 5% CO_2_. Then, the pCDNA-VDAC1-transfected 22RV1 and DU145 cells were used for further analyses, including the MTT assay, apoptosis detection, Western blot, and PCR.

### 4.17. Statistical Analysis

Statistical analysis was performed using GraphPad Prism software (version 9.1.1) with one-way ANOVA analysis. Statistical significance was determined by one-way ANOVA, followed by Tukey’s post hoc test. Differences between groups were considered statistically significant at *p* < 0.05. All experiments were performed in triplicate.

## 5. Conclusions

We isolated and purified the anti-PCa active compound 3,3′-di-O-Me-EA from EHW. Moreover, 3,3′-di-O-Me-EA has favorable drug-like and pharmacokinetic properties. In addition, it could interact with the VDAC1 protein. Furthermore, 3,3′-di-O-Me-EA inhibited 22RV1 and DU145 PCa cell viability and induced apoptosis via up-regulating VDAC1 gene and protein expression levels. Collectively, 3,3′-di-O-Me-EA may be a potential VDAC1-targeted therapeutic agent for PCa treatment.

## Figures and Tables

**Table 1:** The cell viability of the water extract and partition and fraction of Euphorbia humifusa Willd in 22RV-1 PCa cells.

Treatment	Cell Viability (% of Control)
Euphorbia humifusa Willd water extract (780 μg/mL)	68.66 ± 0.40
DCM portion (780 μg/mL)	50.35 ± 0.51
EtOAc (780 μg/mL)	77.18 ± 3.54
n-BuOH (780 μg/mL)	105.15 ± 5.69
Fraction A1 (780 μg/mL)	99.33 ± 12.93
Fraction A2 (780 μg/mL)	86.64 ± 1.91
Fraction A3 (780 μg/mL)	69.17 ± 3.54
Fraction A4 (780 μg/mL)	54.61 ± 1.91
Fraction A5 (780 μg/mL)	37.59 ± 0.82
Fraction A6 (780 μg/mL)	40.57 ± 1.15
Fraction A7 (780 μg/mL)	96.84 ± 0.99
Fraction B1 (780 μg/mL)	70.03 ± 4.95
Fraction B2 (780 μg/mL)	77.00 ± 9.14
Fraction B3 (780 μg/mL)	117.21 ± 4.32
Fraction B4 (780 μg/mL)	39.18 ± 2.24
Fraction B5 (780 μg/mL)	24.91 ± 5.65
Fraction C1 (780 μg/mL)	113.11 ± 3.41
Fraction C2 (780 μg/mL)	93.86 ± 13.49
Fraction C3 (780 μg/mL)	107.55 ± 0.05
Fraction C4 (780 μg/mL)	30.09 ± 4.16
Fraction C5 (780 μg/mL)	11.47 ± 1.72

**Table 2:** Predicted physicochemical, drug-likeness, and ADMET properties of 3,3′-di-O-Me-EA.

Property	Model Name	Predicted Value	Unite
Physicochemical	Canonical SMILES	COc1c(O)cc2c3c1oc(=O)c1c3c(oc2=O)c(c(c1)O)OC	
	MW	330.26	(g/mol)
	Formula	C_16_H_10_O_8_	
	Number of heavy atoms	24	
	Number of aromatic heavy atoms	18	
	No. of H bond acceptor (HBA)	8	
	No. of H bond donor (HBD)	2	
	TPSA (Å)	119.34	
	No. of rotatable bonds	2	
	logP	1.79	
Drug-likeness	Accordance with Lipinski’s rule of Five (RO5)	Yes	Categorical (Yes/No)
	Accordance with Ghose’s rule	Yes	Categorical (Yes/No)
	Accordance with Veber’s rule	Yes	Categorical (Yes/No)
	Accordance with Egan ’s rule	Yes	Categorical (Yes/No)
	Accordance with Muegge ’s rule	Yes	Categorical (Yes/No)
Absorption	Water solubility	−3.01	Numeric (log mol/L)
	Caco2 permeability	−5.75	Numeric (log Papp in 10–6 cm/s)
	Intestinal absorption (human)	82.6	Numeric (% Absorbed)
	P-glycoprotein substrate	NO	Categorical (Yes/No)
	P-glycoprotein I inhibitor	No	Categorical (Yes/No)
	P-glycoprotein II inhibitor	No	Categorical (Yes/No)
	Water solubility	−3.01	Numeric (log mol/L)
Distribution	VDss (human)	−0.69	Numeric (log L/kg)
	Fraction unbound (human)	0.176	Numeric (Fu)
	BBB permeability	−0.393	Numeric (log BB)
Metabolism	CYP2D6 substrate	No	Categorical (Yes/No)
	CYP3A4 substrate	No	Categorical (Yes/No)
	CYP1A2 inhibitor	Yes	Categorical (Yes/No)
	CYP2C19 inhibitor	No	Categorical (Yes/No)
	CYP2C9 inhibitor	Yes	Categorical (Yes/No)
	CYP2D6 inhibitor	No	Categorical (Yes/No)
	CYP3A4 inhibitor	No	Categorical (Yes/No)
Excretion	Total Clearance	Low	Numeric (log ml/min/kg)
	Renal OCT2 substrate	No	Categorical (Yes/No)
toxicity	AMES toxicity	Yes	Categorical (Yes/No)
	hERG I inhibitor	No	Categorical (Yes/No)
	hERG II inhibitor	No	Categorical (Yes/No)
	Skin Sensitization	No	Categorical (Yes/No)
	T.Pyriformis toxicity	1.47	Numeric (log μg/L)

**Table 3:** Interaction profile of the selected active compounds and inhibitors with VDAC1.

Name of Ligand	Name of Protein	PDB ID	Resolution/Å	Average Binding Energy (KJ/mol)	Hydrogen Bonds	Interacting Residues
VDAC1	3,3′-di-O-methyl ellagic acid	6G6U	2.74	−7.5	3	GLN-154, ALA-8, ASN-124
	DIDS			−6.8	2	LYS-96, GLN-154

## Data Availability

The original contributions presented in this study are included in the article/Supplementary Materials. Further inquiries can be directed to the corresponding author.

## Supplementary Materials

The following supporting information can be downloaded at: https://www.mdpi.com/article/10.3390/ph19050652/s1, Figure S1: Dose effects of EHW on the VDAC1 protein expression levels. (A) 22RV-1 cells were treated with EHW (0.01–10μM) for 24 h. (B) DU145 cells were treated with EHW (0.01–10μM) for 24 h. One-way ANOVA was used for the determination of cell viability in apoptotic cell fractions. All experiments were conducted three times (*n* = 3, mean ± SD), and significance was set at * *p* < 0.05 and ** *p* < 0.01. Table S1: Primers used in this study.

## Abbreviations

The following abbreviations are used in this manuscript:

PCa	Prostate cancer
EH	Euphorbia humifusa Willd
EHW	Euphorbia humifusa Willd water extract
3,3′-di-O-Me-EA	3,3′-O-dimethyl ellagic acid
VDAC1	Voltage-dependent anion channel protein 1
SS	Somatostatin
DCM	Dichloromethane
EtOAc	Ethyl Acetate
n-BuOH	n-Butanol
HPLC	High-performance liquid chromatography
HRTOF mass	High-resolution time-of-flight mass spectrometry
NMR	Nuclear magnetic resonance
ADME	Absorption, distribution, metabolism, excretion
SPR	Surface plasmon resonance
Kd	Steady-state affinity
RT-qPCR	Real-time quantitative polymerase chain reaction
WB	Western blot
