# Supplementary material for: 3,3′-Di-O-methylellagic Acid Isolated from Euphorbia humifusa Willd Suppresses Prostate Cancer Cell Viability via Regulating VDAC1 Protein Expression
Source: Pharmaceuticals (Basel). 2026 Apr 22;19(5):652. doi: 10.3390/ph19050652 (PMC13209364; doi:10.3390/ph19050652)
Supplement: Supplementary file 1 [file pharmaceuticals-19-00652-s001.zip › Supplementary Table 1.pdf]

Table 1. Primer used in this study.

Name of gene	Primer Name	Primer Sequence（5'-3'）
β-actin	β actin-F	CCTGGCACCCAGCACAAT
	β actin-R	GGGCCGGACTCGTCATAC
VDAC1	VDAC1-F	GGATGTCTTCACCAAGGGCTATG
	VDAC1-R	GGTGCCTAGTGTATTGTCGGTAT
